# Discriminant Power of Smartphone-Derived Keystroke Dynamics for Mild Cognitive Impairment Compared to a Neuropsychological Screening Test: Cross-Sectional Study

**DOI:** 10.2196/59247

**Published:** 2024-10-30

**Authors:** Jin-Hyuck Park

**Affiliations:** 1 Department of Occupational Therapy College of Medical Science Soonchunhyang University Asan Republic of Korea

**Keywords:** digital biomarker, motor function, digital device, neuropsychological screening, screening tools, cognitive assessment, mild cognitive impairment, keystroke dynamics

## Abstract

**Background:**

Conventional neuropsychological screening tools for mild cognitive impairment (MCI) face challenges in terms of accuracy and practicality. Digital health solutions, such as unobtrusively capturing smartphone interaction data, offer a promising alternative. However, the potential of digital biomarkers as a surrogate for MCI screening remains unclear, with few comparisons between smartphone interactions and existing screening tools.

**Objective:**

This study aimed to investigate the effectiveness of smartphone-derived keystroke dynamics, captured via the Neurokeys keyboard app, in distinguishing patients with MCI from healthy controls (HCs). This study also compared the discriminant performance of these digital biomarkers against the Korean version of the Montreal Cognitive Assessment (MoCA-K), which is widely used for MCI detection in clinical settings.

**Methods:**

A total of 64 HCs and 47 patients with MCI were recruited. Over a 1-month period, participants generated 3530 typing sessions, with 2740 (77.6%) analyzed for this study. Keystroke metrics, including hold time and flight time, were extracted. Receiver operating characteristics analysis was used to assess the sensitivity and specificity of keystroke dynamics in discriminating between HCs and patients with MCI. This study also explored the correlation between keystroke dynamics and MoCA-K scores.

**Results:**

Patients with MCI had significantly higher keystroke latency than HCs (*P*<.001). In particular, latency between key presses resulted in the highest sensitivity (97.9%) and specificity (96.9%). In addition, keystroke dynamics were significantly correlated with the MoCA-K (hold time: *r*=–.468; *P*<.001; flight time: *r*=–.497; *P*<.001), further supporting the validity of these digital biomarkers.

**Conclusions:**

These findings highlight the potential of smartphone-derived keystroke dynamics as an effective and ecologically valid tool for screening MCI. With higher sensitivity and specificity than the MoCA-K, particularly in measuring flight time, keystroke dynamics can serve as a noninvasive, scalable, and continuous method for early cognitive impairment detection. This novel approach could revolutionize MCI screening, offering a practical alternative to traditional tools in everyday settings.

**Trial Registration:**

Thai Clinical Trials Registry TCTR20220415002; https://www.thaiclinicaltrials.org/show/TCTR20220415002

## Introduction

Mild cognitive impairment (MCI), a preclinical stage of Alzheimer disease (AD), causes cognitive declines, especially in memory and executive functions beyond what is expected from normal aging [1]. The importance of early intervention for patients with MCI has highlighted the need for continuous testing of patients with MCI to ensure they have not transitioned from MCI to AD [2]. Current test methods mainly depend on neuropsychological screening tools implemented in a clinical setting [3-5]. Unfortunately, previous studies have reported the low sensitivity of these tools, particularly the Montreal Cognitive Assessment (MoCA), which limits their effectiveness in discriminating early MCI from healthy aging [4-6]. In addition, various neuropsychological screening tools, such as the Rapid Cognitive Screen, Six-item Screener, Mini-Cog, and Clock Drawing Test, were investigated to determine their usefulness in screening for MCI. However, similar to the MoCA, these tools were found to have low sensitivity and specificity in detecting MCI, leading to limitations in their application. Although tools such as the Short Test of Mental Status and Memory Alteration Test have been reported to show high sensitivity and specificity, only one study has examined these tools [7], which leaves their effectiveness uncertain. Therefore, it has been concluded that existing neuropsychological screening tools that evaluate cognitive function alone are ineffective for screening MCI [7-9].

Apart from cognitive declines, there are several indications that people with MCI are associated, to a certain degree, with motor dysfunction in upper-extremity functions [10]. A previous study indicated that individuals with MCI have poor performance in dual-task gait tests, which involve a sensor-based, upper-extremity motor task during cognitive testing. This could be a marker to distinguish MCI from normal aging, with quite high sensitivity (over 80%) and specificity (over 70%) [11]. Several studies suggest that MCI markers, especially digital biomarkers using data derived from mobile and wearable devices, could be a promising new research field, along with the sensitivity and usability of sensors [11,12]. From this perspective, variations in typing on and touching a smartphone screen have been investigated for early MCI screening, showing considerable differences compared to healthy aging [13,14].

Smartphone-induced behaviors are significantly correlated with cognitive performance, such as the temporal characteristics of typing speed, removal rate, and interval time between pushing and releasing buttons. These keystroke dynamics have been identified as a potential marker for cognitive decline, particularly in attention and working memory [12,15,16]. Keystroke dynamics could be collected seamlessly as users interact with their smartphones during their daily routines, capturing their natural behavior and thereby offering high ecological validity. Therefore, keystroke dynamics have been used for the early screening of AD and Parkinson disease [14,17]. In a previous study, features of keystroke dynamics extracted from text-typing activities were investigated to monitor signs of early cognitive decline in MCI, showing their clinical efficacy [12,15,17]. Especially, the discriminant power of these features could be enhanced when combined with conventional features of cognitive decline such as memory and linguistic abilities [17]. These findings suggest the potential for multimodal features, indicating that this approach is needed to discriminate MCI.

However, most previous studies on sensor-based markers to discriminate MCI examined their efficacy by combining sensor-derived data that could be difficult to use and assess, such as those from accelerometers, voice recorders, and motion capture sensors. This approach requires special devices that are not typically used and must be installed on the body or in the home, creating an artificial environment that deviates from the subjects’ natural state and reduces ecological validity, resulting in findings that are not applicable to everyday living [7,18,19]. Also, even if some prior studies revealed the potential of smartphone-derived markers for MCI, it is difficult to ensure their clinical applicability as no direct comparisons have been made with widely used screening tools [13-15,17].

Therefore, this study aimed to examine the efficacy of digital biomarkers obtained through routine interactions with a smartphone keyboard to distinguish MCI from healthy aging in a nonclinical setting, taking into consideration the pragmatic condition of everyday living. In addition, this study aimed to investigate whether classification performance could be improved through the combination of keystroke dynamics and conventional screening tools.

## Methods

### Participants

The participants were recruited from senior centers and daycare centers in Seoul and Asan, South Korea. The study included 2 groups: 64 healthy controls (HCs) and 47 patients with MCI. In accordance with a previous study [1], the inclusion criteria for MCI were as follows: (1) a subjective memory complaint; (2) memory impairment relative to age- and education-matched HCs, confirmed by performance on a neuropsychological battery (below 1.5 SD); (3) intact global cognitive function, confirmed by a score on the Korean version of the Mini-Mental Status Examination (MMSE-K); (4) independent activities of daily living; and (5) experience using a smartphone for at least 3 months. The exclusion criteria were as follows: (1) diagnosed with dementia by a clinician, (2) neurological or psychiatric disorders such as stroke and depression, and (3) visual or auditory impairments. These criteria are based on the original Petersen criteria, which restricts MCI to memory problems (amnestic MCI) only.

### Ethical Consideration

This study was approved by the Institutional Review Board of Soonchunhyang University (202306-SB-070-04) and registered at the Thai Clinical Trials Registry (TCTR20220415002). This study adhered to all relevant guidelines and regulations concerning the ethical conduct of human subject research. All participants provided written informed consent prior to the initiation of any study activities. The consent process ensured that all participants were fully informed about the nature of the study, its objectives, and their rights as participants, including the voluntary nature of their participation. To protect participants’ privacy and confidentiality, all study data were anonymized and deidentified before analysis. No identifying information was collected or stored that could be traced back to individual participants. The study followed strict data protection protocols to ensure the confidentiality of the information provided by the participants. All participants were compensated with ₩30,000 (~US $21.74) for their study participation. No images that could potentially identify individual participants were included in the paper or supplementary materials.

### Procedure

All participants performed the Korean version of the MoCA (MoCA-K) and the computerized Corsi block-tapping test (CBT) before their keystroke data were collected. Both the MoCA-K and the computerized CBT were used to assess baseline cognitive performance. Subsequently, the relationship between these assessments and keystroke dynamics was examined. The MoCA-K was selected for its role as a screening tool for MCI and its ability to assess global cognitive function, while the CBT was chosen for its efficacy in assessing working memory decline, which is common in patients with MCI.

Participants then installed a custom-developed mobile app that allows users to measure health conditions through typing on a smartphone. The app keyboard replaced the default keyboard, and participants were allowed to become accustomed to it for a week. During regular typing activities, keyboard interactions (keystroke dynamics) such as key presses and releases were recorded and time-stamped in the background. The keystroke dynamics were stored in a JSON format and indexed in a database, accessible only to the app. The app periodically delivered information on uniquely coded keystroke dynamics to a remote cloud server (Microsoft Azure), when the participant’s device was connected to Wi-Fi and charging.

**Figure 1:**
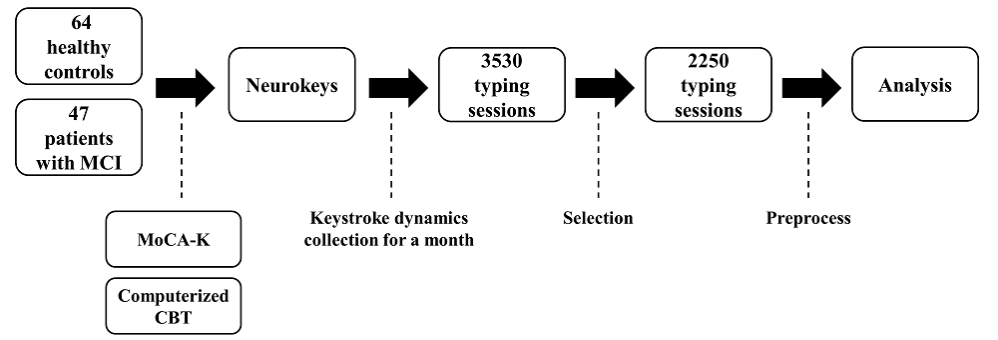
The flow diagram of the procedure of this study. CBT: Corsi block-tapping test; MCI: mild cognitive impairment; MoCA-K: Korean version of Montreal Cognitive Assessment.

Data collection lasted a month, and all participants were instructed not to have anyone other than themselves type on their phones. Although it was impossible to confirm whether participants typed all data definitively, the author reconfirmed that only the participants themselves used their phones for 1 month before data analysis. This study did not impose a minimum daily usage requirement on participants, as the aim of this study was to capture keystroke dynamics that most accurately reflect the natural status of their daily lives. Within a month, 3530 typing sessions were collected, 2250 (63.7%) from 64 HCs and 1280 (36.3%) from 35 patients with MCI. Of the 3530 typing session, 2740 (77.6%) with more than 40 key presses per session were finally used to ensure sufficient data for meaningful comparison, in accordance with a previous study [14]. The flow diagram of the overall procedure of this study is presented in Figure 1.

**Figure 2:**
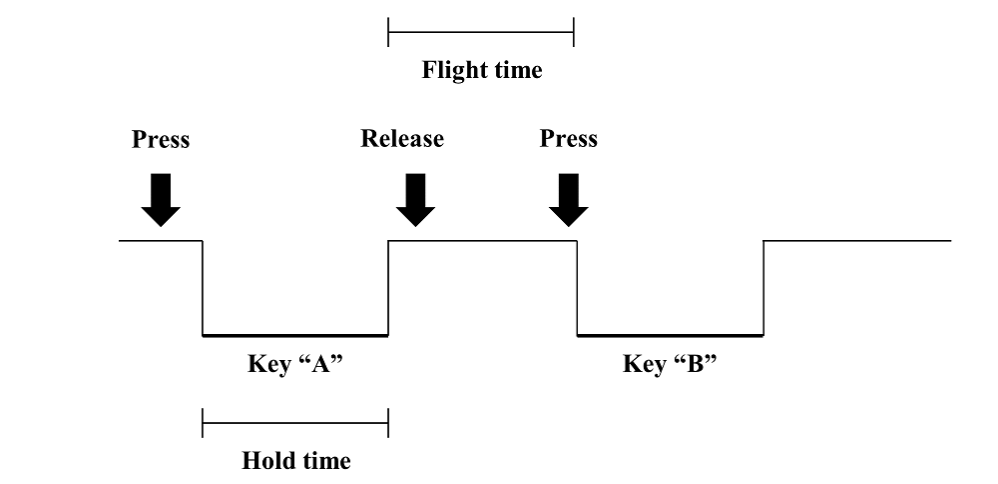
Schematic representation of keystroke dynamics derived from time-stamped keyboard interactions.

Two variables of the keystroke dynamics stored in the JSON files were analyzed in this study: hold time (HT) and flight time (FT). These 2 variables were chosen because a previous study showed that they are significant in discriminating MCI from healthy aging among other keystroke dynamics [13]. HT refers to the time interval between pressing and releasing a key, while FT indicates the time interval between releasing a key and pressing the next key (Figure 2). To preprocess data, HT >700 microseconds and FT >3 seconds were excluded according to the guidelines in a previous study [14].

### Outcome Measures

The original MoCA was developed to discriminate MCI from normal aging, and its domains consist of visuospatial/executive function, attention, memory, language, abstraction, and orientation [20]. Its score ranges from 0 to 30 points, where higher scores indicate better global cognitive function. The MoCA-K was adapted based on the original MoCA [21] (Multimedia Appendix 1). The cutoff score of the MoCA-K for MCI was 23, and 1 point could be added for participants with fewer than 6 years of education. In a previous study with a cutoff score of 23, its sensitivity and specificity were 94.2% and 40.5%, respectively [21].

The computerized CBT was used to assess attention and working memory. Since patients with MCI commonly exhibit a decline in memory and executive functions [1], a working memory test that evaluates both memory and executive function components was chosen [22]. In the task, 9 white squares were randomly located on a tablet monitor. Some of the squares changed their color from white to red in a sequential manner while participants were asked to memorize the locations and sequences of the changed squares. Afterward, participants were instructed to point out the changed squares in the order in which their colors had changed by touching the screen [23,24]. In this study, the number of squares to be changed was 5, and 15 trials were implemented. A previous study indicated that the average square span of HCs is 5 [23].

The MMSE is a widely used tool for screening cognitive impairment. The MMSE-K was the result of the MMSE being translated into Korean and standardized [25] (Multimedia Appendix 2). It comprises 12 items assessing 7 cognitive domains: orientation, memory registration, attention and calculation, recall, language, understanding and judgment, and visual construction. Its score ranges from 0 to 30, with scores below 24 indicating cognitive impairment [25].

All outcome measures were implemented by an occupational therapist with 5 years of clinical experience.

### Statistical Analysis

SPSS for Windows (version 22.0; IBM Corp) was used to analyze the data. The demographic characteristics of the participants were analyzed using descriptive statistics. Independent 2-tailed *t* test and chi-square test were used to compare both groups. A receiver operating characteristic (ROC) curve analysis was used to confirm sensitivity and specificity, and a cutoff score for patients with MCI was determined according to the highest Youden Index (*sensitivity + specificity – 1*), which could be a criterion for choosing an optimal cutoff score. To conduct ROC curve analysis of the combination of keystroke dynamics as a single variable, predictive probability through logistic regression was used in accordance with a previous study [26]. A Spearman correlation was performed to examine the relationship between keystroke dynamics and global cognitive function and working memory. Statistical significance was set at *P*<.05.

## Results

### General and Clinical Characteristics in Both Groups

There were no statistically significant differences in demographic characteristics between both groups (all *P*>.05) except for the score of the MoCA-K (*P*<.001). This finding indicated that HCs were age- and education-matched to patients with MCI except for cognitive function (Table 1).

**Table 1:** General and clinical characteristics of participants (N=111).

Characteristics		HCs^a^ (n=64)	Patients with MCI^b^ (n=47)	*t* test or chi-square test (*df*)		*P* value	
**Age (y), mean (SD)**		75.52 (7.00)	74.45 (6.98)	.795 (109)^c^		.43	
**Sex, n (%)**					.053 (1)^d^		.82
	Male	30 (47)	21 (45)				
	Female	34 (53)	26 (55)				
**Education period (y), mean (SD)**		6.48 (4.41)	6.34 (4.81)	.163 (109)^c^		.87	
**Typing experience (y), mean (SD)**		11.41 (1.25)	11.87 (1.81)	1.600 (109)^c^		.11	
**Grooved pegboard test (s), mean (SD)**							
	Preferred hand	82.98 (5.81)	82.28 (6.02)	0.624 (109)^c^		.53	
	Nonpreferred hand	92.25 (5.48)	91.15 (4.90)	1.097 (109)^c^		.28	
**MMSE-K^e^ (point), mean (SD)**		26.86 (1.39)	26.55 (1.15)	1.229 (109)^c^		.22	
**MoCA-K^f^ (point), mean (SD)**		25.83 (2.11)	22.77 (2.26)	7.316 (109)^c^		<.001	
**CBT^g^ (accuracy), mean (SD)**		.773 (0.06)	.687 (0.07)	6.817 (109)^c^		<.001	
**HT^h^ (ms), mean (SD)**		108.77 (25.25)	184.85 (25.24)	15.689 (109)^c^		<.001	
**FT^i^ (ms), mean (SD)**		622.925 (135.14)	1351.51 (242.75)	20.151 (109)^c^		<.001	

^a^HC: health control.

^b^MCI: mild cognitive impairment.

^c^2-tailed *t* test.

^d^Chi-square test.

^e^MMSE-K: Korean version of the Mini-Mental State Examination.

^f^MoCA-K: Korean version of the Montreal Cognitive Assessment.

^g^CBT: Corsi block-tapping test.

^h^HT: hold time.

^i^FT: flight time.

### Sensitivity, Specificity, and Discriminant Power

**Figure 3:**
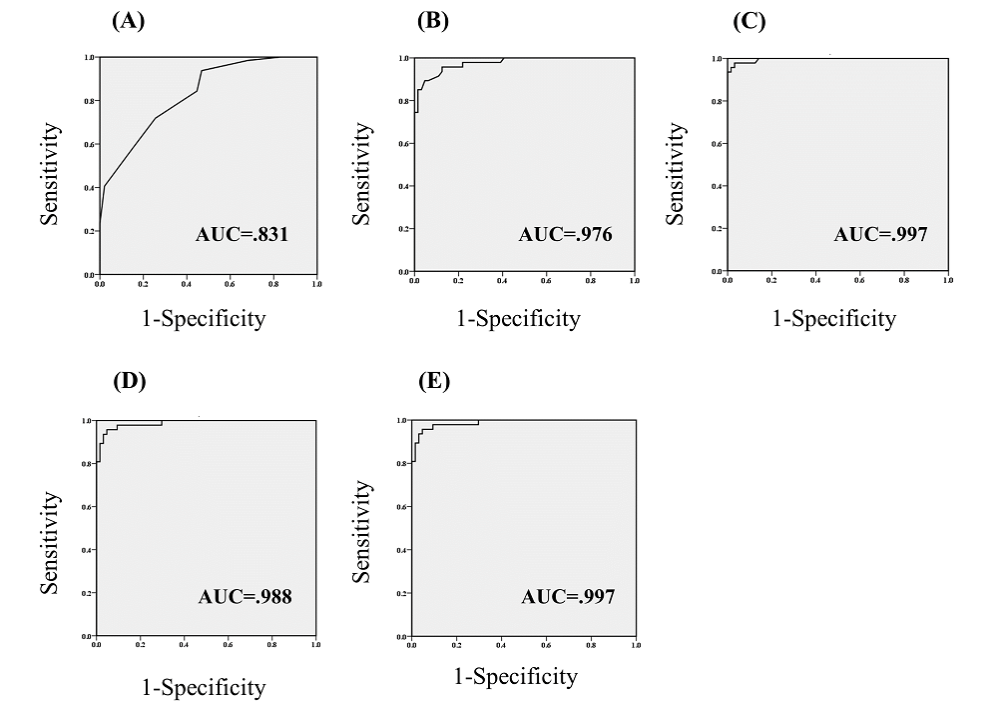
ROC curves of 5 predictors: (A) MoCA-K, (B) HT, (C) FT, (D), MoCA-K + HT, and (E) MoCA-K + FT. Greater AUC values indicate higher power in discriminating patients with MCI from healthy controls. AUC: area under the curve; FT: flight time; HC: healthy control; HT: hold time; MCI: mild cognitive impairment; MoCA-K: Korean version of Montreal Cognitive Assessment; ROC: receiver operating characteristic.

For discriminating patients with MCI from the matched HC, both HT and FT showed a higher Youden Index than the MoCA-K (HT: .847; FT: .947; and MoCA-K: .469), suggesting that keystroke dynamics can better discriminate MCI compared to the conventional MCI screening tool (Table 2). Specifically, FT yielded a maximum sensitivity (97.9%) and specificity (94.7%). Interestingly, when combined with the MoCA-K, HT had a higher Youden Index, while FT did not (Table 2 and Figure 3).

**Table 2:** Sensitivity and specificity of MCI^a^ detection (N=111).

Variable	Sensitivity	Specificity	Youden index	Cutoff	AUC^b^ (95% CI)	*P* value
MoCA-K^c^ (point)	.938	.532	.469	22.50	.831 (.756-.903)	<.001
HT^d^ (ms)	.894	.953	.847	159.50	.976 (.954-1.000)	<.001
FT^e^ (ms)	.979	.969	.947	884.00	.997 (.990-.1000)	<.001
MoCA-K + HT (probability)	.957	.953	.911	.427	.988 (.973-.1000)	<.001
MoCA-K + FT (probability)	.979	.969	.947	.255	.997 (.990-.1000)	<.001

^a^MCI: mild cognitive impairment.

^b^AUC: area under the curve.

^c^MoCA-K: Korean version of the Montreal Cognitive Assessment.

^d^HT: hold time.

^e^FT: flight time.

### Correlation in Keystroke Dynamics and Cognitive Function

Keystroke dynamics were found to be significantly correlated with the MoCA-K (HT: *r*=–.468; *P*<.001; FT: *r*=–.491; *P*<.001) and the CBT (HT: *r*=–.487; *P*<.001; FT: *r*=–.492; *P*<.001; Table 3). These findings suggested that keystroke dynamics are associated with global cognitive function and working memory.

**Table 3:** Correlation of cognitive function with keystroke dynamics.

			MoCA-K^a^		CBT^b^		HT^c^		FT^d^
**MoCA-K**									
	*r*	1		.302		–.468		–.491	
	*P* value	—^e^		.001		<.001		<.001	
**CBT**									
	*r*	.302		1		–.487		–.492	
	*P* value	.001		—		<.001		<.001	
**HT**									
	*r*	–.468		–.487		1		.733	
	*P* value	<.001		<.001		—		<.001	
**FT**									
	*r*	–.491		–.492		.733		1	
	*P* value	<.001		<.001		<.001		—	

^a^MoCA-K: Korean version of the Montreal Cognitive Assessment.

^b^CBT: Corsi block-tapping test.

^c^HT: hold time.

^d^FT: flight time.

^e^Not applicable.

## Discussion

### Principal Findings

The aim of this study was to evaluate keystroke dynamics as potential digital biomarkers for MCI. For this purpose, keystroke dynamics were investigated in 64 HCs and 47 patients with MCI, alongside the conventional screening tool. By measuring keystroke dynamics through the natural spontaneous use of smartphones, this study aimed to identify distinct patterns that differentiate HCs and patients with MCI.

The findings of this study revealed that keystroke dynamics, particularly HT and FT, were effective in distinguishing between HCs and patients with MCI. Notably, the sensitivity and specificity of keystroke dynamics surpassed those of the conventional screening tool. Furthermore, FT showed the highest Youden Index for all outcome measures, and its Youden Index did not increase when combined with the MoCA-K, suggesting that FT can effectively distinguish MCI on its own; this finding highlights its potential as an alternative screening method for MCI.

### Comparison to Prior Work

MCI-induced dysfunction includes motor as well as cognitive dysfunction; this finding is supported by several studies reporting that the degree of motor decline might help in dissociating MCI from healthy aging [27]. Specifically, dominant hand bradykinesia could be an efficient marker to distinguish MCI [14]. In addition, a previous study indicated that Parkinson disease signs characterized by slow movement are correlated with patients with MCI. The findings of this study, showing longer HT and FT in patients with MCI, are consistent with these studies, emphasizing the link between motor function decline and MCI [28]. Notably, motor impairment in patients with MCI considerably affects fine motor skills more than gross motor skills, which leads to a decline in dexterity skills in the upper limbs, including typing [29,30].

Declines in motor function could be attributed to working memory deficits [31,32]. Working memory is required to maintain motor chunk length in high-level motor sequence performance. However, patients with MCI, even patients with amnestic MCI, are typically characterized by deficits in working memory due to reduced brain volume in the prefrontal cortex [33]. This aligns with this study’s findings, showing lower performance in the CBT among patients with MCI compared to HCs. Interestingly, no statistically considerable difference in hand dexterity between both groups was confirmed by the grooved pegboard test in this study, suggesting that longer HT and FT in individuals with MCI are more likely to be attributed to a deficit in working memory rather than a purely motor-related issue. Therefore, smartphone typing in individuals with MCI could be negatively affected by working memory deficits.

Prior studies reported the potential of keystroke dynamics derived from touchscreen typing to capture MCI, which supports the findings of this study [13-15]. However, these studies did not compare keystroke dynamics to traditional screening tools, which limits the practical clinical applicability of keystroke dynamics [13-16]. In contrast, this study demonstrated that both HT and FT showed a greater discriminant power for MCI than the MoCA-K, suggesting that keystroke dynamics could be more beneficial to discriminate MCI from normal aging than conventional screening tools. The main factor underlying this comparative finding is based on the metrics between the keystroke dynamics and the MoCA-K. Delayed HT and FT could represent a working memory deficit, one of the hallmarks of MCI [33]. On the other hand, the MoCA-K includes orientation and language domains, which are not obviously impaired in MCI as it represents global cognitive function [5,6]. This difference in the metric could explain the superiority of the keystroke dynamics for MCI screening.

Notably, FT showed the highest Youden Index for all outcome measures, and its Youden Index did not increase when combined with the MoCA-K. This result suggests that FT is more useful in distinguishing MCI than HT. Furthermore, delayed FT can be used alone as a good marker of MCI, which indicates that a keystroke dynamics monitor could be a cost-effective way of screening MCI, considering that it does not require the additional implementation of neuropsychological tests to increase screening accuracy. Similarly, in a previous study, an ensemble model combining keystroke dynamics and linguistic features did not show superiority in discriminating MCI over keystroke dynamics alone [14], supporting the findings of this study. On the other hand, recent studies have expanded keystroke dynamics analysis by incorporating time feature analysis with graphical transformations analyzed using convolutional neural networks. Additionally, techniques such as natural language processing (eg, noun-to-verbs ratio) have been applied [15]. There have also been efforts to combine keystroke dynamics with brain imaging data analyzed through machine learning to enhance discriminant accuracy [34]. This reflects a growing interest in multimodal biomarkers of naturalistic behavioral data and machine learning methods, integrating various data sources to improve the robustness of biometric systems [35].

### Strengths

One of the main strengths of this study is the naturalistic collection of data in an unobtrusive way, reflecting the natural status of the participants while showing a significant correlation with the clinical screening tool. Digital health is an emerging field that enhances disease detection and management through the objective capturing of behavioral characteristics in natural situations [17]. From this digital health perspective, keystroke dynamics derived from the natural interaction with smartphones could be key factors in digital health for individuals with MCI. In addition, keystroke dynamics could be noninvasively and longitudinally captured during routine typing in real daily life without any additional effort, ensuring long-term adherence. Long adherence plays a key role in a continuous process of monitoring, diagnosis, and treatment, which is of high importance toward personalized management [14]. Considering that individualized tests that ignore the longitudinal aspect of data collection could miss people with cognitive impairment in real-world settings [36], digital biomarkers from daily smartphone use could ensure that MCI is not missed, allowing for early intervention, compared to conventional screening tools that are periodically implemented in clinics.

### Limitations and Future Directions

Despite the promising findings and strengths of this study, there are some limitations to be considered. First, these findings could not be generalized as MCI was limited to its amnestic type. Nevertheless, considering that individuals with amnestic MCI show less heterogeneity in cognitive function than those with nonamnestic MCI and multidomain MCI [5], the findings of this study have clinical implications. Second, although working memory decline in participants with MCI was investigated via the CBT, the prefrontal cortex, which underlies working memory, was not objectively observed. Thus, it is difficult to affirm whether changes in keystroke dynamics in people with MCI are due to working memory deficits caused by neurodegeneration in the prefrontal cortex. A neuroimaging study will provide more objective evidence of changes in the keystroke dynamics of patients with MCI, allowing for subgroup analysis based on neurodegeneration severity. Third, although this study confirmed through subjective reports that participants did not allow others to use their smartphones, this was not objectively verified. Finally, this study demanded technology familiarization with a smartphone to some extent.

In the future, therefore, to develop an ecologically valid MCI screening variable for a larger population, it is necessary to investigate activities that are easier to use and more widely available. Additionally, it is necessary to determine if different MCI subtypes exhibit distinct features of keystroke dynamics and incorporate these findings into brain imaging data to establish multimodal biomarkers.

### Conclusion

Keystroke dynamics reflecting both motor and cognitive deficits were identified as a more clinically useful digital biomarker for MCI, compared to a neuropsychological screening tool. The findings of this study present a new perspective on detecting MCI via keystroke dynamics from smartphone use. These promising results suggest that measures from smartphone typing could serve as an ecologically valid digital biomarker in a nonclinical setting, acting as a surrogate for laboratory-based neuropsychological screening tools.

## Appendix

Multimedia Appendix 1 Korean version of the Montreal Cognitive Assessment (MoCA-K) form.

Multimedia Appendix 2 Korean version of the Mini-Mental Status Examination (MMSE-K) form.

## Abbreviations

AD: Alzheimer disease
CBT: Corsi block-tapping test
FT: flight time
HC: healthy control
HT: hold time
MCI: mild cognitive impairment
MMSE: Mini-Mental Status Examination
MMSE-K: Korean version of the Mini-Mental Status Examination
MoCA: Montreal Cognitive Assessment
MoCA-K: Korean version of the Montreal Cognitive Assessment
ROC: receiver operating characteristic

## Data Availability

The datasets generated during and/or analyzed during this study are available from the corresponding author on reasonable request.
