# Correction: Discriminant Power of Smartphone-Derived Keystroke Dynamics for Mild Cognitive Impairment Compared to a Neuropsychological Screening Test: Cross-Sectional Study

**DOI:** 10.2196/86291

**Published:** 2025-11-07

**Authors:** Jin-Hyuck Park

**Affiliations:** 1 Department of Occupational Therapy College of Medical Science Soonchunhyang University Asan Republic of Korea

In “Discriminant Power of Smartphone-Derived Keystroke Dynamics for Mild Cognitive Impairment Compared to a Neuropsychological Screening Test: Cross-Sectional Study” [1], the author noted one error and made one clarification.

During manuscript preparation, a generative artificial intelligence (AI)–based language editing tool (ChatGPT-4, OpenAI) was used solely to smoothly refine the language and improve clarity. The tool was not used to generate or modify any scientific content, data, or results. However, during this process, an inadvertent substitution error was introduced in the Methods section, which incorrectly described the use of the “Neurokeys” mobile app (Neurocast). In fact, the study utilized a custom-developed mobile app that was independently developed by the author. No data, software, or technology from Neurocast was used.

The corresponding text in the Methods section has been corrected as a result. The following text:

Participants then installed the “Neurokeys” mobile app (Neurocast), developed for Android and iOS, which is available for free and allows users to measure health conditions through typing on a smartphone. The Neurokeys keyboard replaced the default keyboard, and participants were allowed to become accustomed to it for a week.

has been revised to:

Participants then installed a custom-developed mobile app that allows users to measure health conditions through typing on a smartphone. The app keyboard replaced the default keyboard, and participants were allowed to become accustomed to it for a week.

This correction clarifies that the mobile app used in the study was independently developed by the author and not associated with Neurocast. The correction does not affect the results, analyses, or conclusions of the paper.

The correction will appear in the online version of the paper on the JMIR Publications website, together with the publication of this correction notice. Because this was made after submission to PubMed, PubMed Central, and other full-text repositories, the corrected article has also been resubmitted to those repositories.
