# Supplementary material for: Discriminant Power of Smartphone-Derived Keystroke Dynamics for Mild Cognitive Impairment Compared to a Neuropsychological Screening Test: Cross-Sectional Study
Source: J Med Internet Res. 2024 Oct 30;26:e59247. doi: 10.2196/59247 (PMC11561447; doi:10.2196/59247)
Supplement: Multimedia Appendix 1 [file jmir_v26i1e59247_app1.pdf]

한국판 몬트리올 인지평가 (K-MOCA)

이 름:
교육수준: 생년월일:
성 별: 실시일:

시공간 / 집행기능		시계 그리기 (열한시 십분) (3점)	점수
	육면체 그리기		

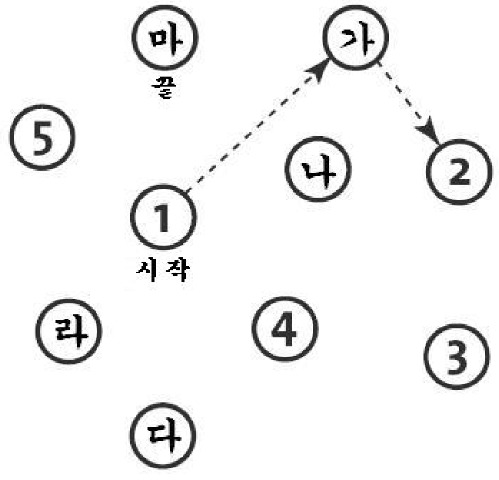

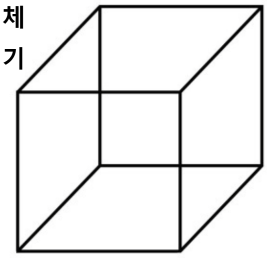

[ ] [ ] [ ] [ ] [ ] ___/5

윤곽 숫자 바늘

이름대기

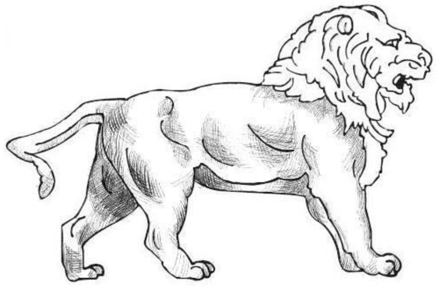

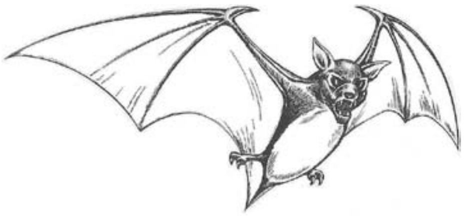

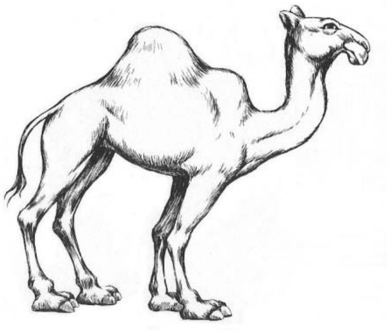

[ ] [ ] [ ] ___/3

기억력		얼 굴	비 단	학 교	피 리	노 랑	점수 없음
5개 단어를 모두 들은 후 즉각회상 실시. 1차 시행을 성공하였더라도 2회 모두 실시함. 5분 후에 지연회상 실시.	1차 시행						
	2차 시행						

**주의력** 1초에 1개씩 숫자를 읽어 줌.	순서대로 따라 외우기 [ ] 2 1 8 5 4<br>거꾸로 따라 외우기 [ ] 7 4 2	___/2
쓰인 순서대로 요일을 불러 줌. 피검자는 "월"에만 손뼉을 쳐야 함. 2개 이상의 오류는 0점<br>[ ] 토 화 월 수 일 화 월 월 목 금 토 화 월 토 월 금 목 금 월 월 월 목 월 일 화 토 월 월 화		___/1
100에서 7씩 빼기 [ ] 93 [ ] 86 [ ] 79 [ ] 72 [ ] 65<br>*정답이 4 또는 5개: **3점**, 2 또는 3개: **2점**, 1개: **1점**, 0개: **0점**		___/3
**언 어** 따라 말하기: 칼날같이 날카로운 바위. [ ] 스물 일곱 개의 찬 맥주병이 냉장고에 있다. [ ]		___/2
유창성 / "ㄱ"으로 시작되는 단어를 1분 동안 최대한 많이 말씀해 주세요. [ ] ______ (N ≥ 6개 단어)		___/1
**추상력** 공통성 (예) 사과 – 복숭아 = 과일 [ ] 기차 – 비행기 [ ] 시계 – 저울		___/2

지연회상	회상 단어	얼 굴	비 단	학 교	피 리	노 랑	단서 없이 회상한 단어에만 점수를 줌.	___/5
	단서 없음	[ ]	[ ]	[ ]	[ ]	[ ]		
선택 사항	범주 단서							
	선다형 단서							

지남력 [ ] 일 [ ] 월 [ ] 년 [ ] 요일 [ ] 장소 [ ] 도시 이름 ___/6

© Z. Nasreddine MD Version 7.1 www.mocatest.org 규 준: 강연욱 등(2009) 참조

검 사 자: ________________

총 점 _____ / 30
