# Supplementary material for: Discriminant Power of Smartphone-Derived Keystroke Dynamics for Mild Cognitive Impairment Compared to a Neuropsychological Screening Test: Cross-Sectional Study
Source: J Med Internet Res. 2024 Oct 30;26:e59247. doi: 10.2196/59247 (PMC11561447; doi:10.2196/59247)

K-MMSE (Mini-Mental State Exam)

항목			K-MMSE
지남력-시간 (5점)	년		0 1
	월		0 1
	일		0 1
	요일		0 1
	계절		0 1
지남력-장소 (5점)	나라		0 1
	시.도		0 1
	무엇하는 곳		0 1
	현재 장소명		0 1
	몇층		0 1
기억등록 (5점)	비행기		0 1
	연필		0 1
	소나무		0 1
주의집중 및 계산 (5점)	100 -7		0 1
	-7		0 1
	-7		0 1
	-7		0 1
	-7		0 1
기억회상 (3점)	비행기		0 1
	연필		0 1
	소나무		0 1
언어 및 시공간구성능력 (9점)	이름대기	시계	0 1
		볼펜	0 1
	명령시행	종이를 뒤집어	0 1
		반으로 접은 다음	0 1
		저에게 주세요	0 1
	따라말하기	“백문이 불여일견”	0 1
	읽기		0 1
	쓰기		0 1
	오각형		0 1
**총점**	**/30**		

눈을 감으세요.

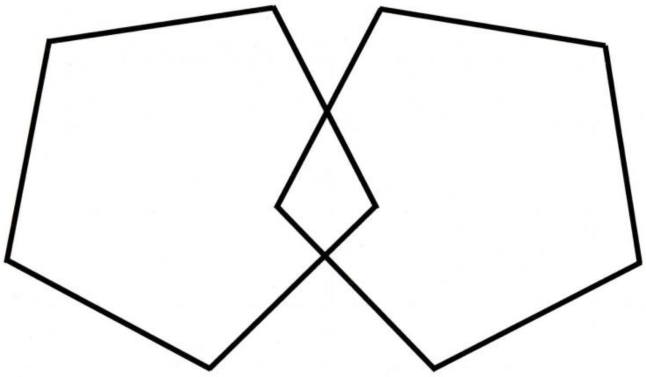

Supplement: Multimedia Appendix 2 [file jmir_v26i1e59247_app2.pdf]
